# Variable Nitrogen Fixation in Wild *Populus*

**DOI:** 10.1371/journal.pone.0155979

**Published:** 2016-05-19

**Authors:** Sharon L. Doty, Andrew W. Sher, Neil D. Fleck, Mahsa Khorasani, Roger E. Bumgarner, Zareen Khan, Andrew W. K. Ko, Soo-Hyung Kim, Thomas H. DeLuca

**Affiliations:** 1 School of Environmental and Forest Sciences, College of the Environment, University of Washington, Seattle, Washington, United States of America; 2 Department of Microbiology, University of Washington, Seattle, Washington, United States of America; Umeå Plant Science Centre, Umeå University, SWEDEN

## Abstract

The microbiome of plants is diverse, and like that of animals, is important for overall health and nutrient acquisition. In legumes and actinorhizal plants, a portion of essential nitrogen (N) is obtained through symbiosis with nodule-inhabiting, N_2_-fixing microorganisms. However, a variety of non-nodulating plant species can also thrive in natural, low-N settings. Some of these species may rely on endophytes, microorganisms that live within plants, to fix N_2_ gas into usable forms. Here we report the first direct evidence of N_2_ fixation in the early successional wild tree, *Populus trichocarpa*, a non-leguminous tree, from its native riparian habitat. In order to measure N_2_ fixation, surface-sterilized cuttings of wild poplar were assayed using both ^15^N_2_ incorporation and the commonly used acetylene reduction assay. The ^15^N label was incorporated at high levels in a subset of cuttings, suggesting a high level of N-fixation. Similarly, acetylene was reduced to ethylene in some samples. The microbiota of the cuttings was highly variable, both in numbers of cultured bacteria and in genetic diversity. Our results indicated that associative N_2_-fixation occurred within wild poplar and that a non-uniformity in the distribution of endophytic bacteria may explain the variability in N-fixation activity. These results point to the need for molecular studies to decipher the required microbial consortia and conditions for effective endophytic N_2_-fixation in trees.

## Introduction

The microbiota of plants can provide a wide range of benefits to the host including increased tolerance to drought, salt, or temperature extremes, the production of phytohormones, resistance to microbial pathogens, detoxification of pollutants, and increased nutrient acquisition [1–4]. In nutrient-limiting environments, plants are most likely to form associations with microorganisms capable of fixing atmospheric dinitrogen (N_2_) gas into usable forms. Bacteria accomplish this reduction through the action of nitrogenase, a complex multi-subunit enzyme that is inhibited by oxygen. In both aerobic and microaerobic environments, however, diazotrophic (N_2_-fixing) microorganisms can use a variety of strategies to protect the nitrogenase complex [5,6].

In the well-known symbioses of rhizobia with legumes and *Frankia* with actinorhizal plants, diazotrophic bacteria fix N_2_ while within specialized root nodule structures where oxygen levels are regulated. For non-nodulating plants, however, recent evidence points to symbiosis with internal microorganisms, termed endophytes, as a mechanism for these plants to obtain their essential nitrogen (N) [7]. Although N_2_-fixing endophytes are not in an external structure, the colonized plant tissue itself may be microaerobic [8,9]. The expression of the nitrogenase subunit genes is regulated by oxygen [10], and since *nifH* expression has been demonstrated in several systems while the bacteria are associated with the plant [11–14], the appropriate conditions must therefore have been met.

N_2_-fixing endophytes have been isolated from such varied species as kallar grass [15], sugarcane [16], wild rice [17,18], maize [19], *Sorghum halepense* [20], miscanthus [21], elephant grass [22], rock-dwelling cactus [23], sweet potato [24,25], Boreal mosses [26], dune grasses [27], coffee plants [28], and conifers [29,30], N_2_-fixation was directly quantified in the Graminaceae such as sugarcane, wheat, and rice [13,15,18,31]. Several field studies utilized the ^15^N natural abundance technique to demonstrate very substantial inputs of N in sugarcane and elephant grass through biological N_2_-fixation [32–34]. Despite the decades of research on endophytic N_2_-fixation, the idea that significant symbiotic N_2_-fixation can occur in plant tissue without root nodules has remained controversial [35]. In a recent seminal paper by Pankievicz, et al., symbiotic N_2_-fixation by endophytic and associative bacteria of sugarcane was unequivocally demonstrated in a model C4 grass system [36]. Addition of diazotrophic bacteria to *Setaria viridis* resulted in N transfer as demonstrated by ^13^N tracer studies, relief of N-stress symptoms, and restoration of the metabolic profile to that of an N-sufficient state. There is a need for more studies utilizing these technically-challenging and expensive yet direct assays of N_2_-fixation using isotope tracers.

Although there are numerous reports on biological N_2_-fixation (BNF) by endophytes in the Graminaceae, especially of tropical grasses, few have reported BNF in temperate plants, especially in trees [29,37]. Members of the Salicaceae family, including poplars (*Populus* sp.) and willows (*Salix* sp.), are early successional tree species able to colonize nutrient-poor environments, and are increasingly important for bioenergy, wood products, and environmental services [38,39]. Several studies have assessed the microbiome of *P*. *deltoides* and hybrid poplar, and its involvement in poplar growth promotion and remediation of pollutants [40–44]. *Populus trichocarpa* can thrive in riparian zones where regular flooding from high alpine snow melt deposits cobble and sand, creating new substrate for colonization [45]. Due to its rocky composition, however, this new substrate can be nutrient-limited. The ability to colonize such substrate has often been attributed to N use efficiency; however, in these nutrient-limited areas devoid of organic matter, there is little organic N with which to be efficient. We previously reported the presence of endophytic *Rhizobium tropici* [46] from hybrid poplar grown in greenhouses. A variety of other diazotrophic endophytic species were subsequently isolated from wild poplar growing in a natural riparian area dominated by cobble [47]. These endophytes were shown to be mutualistic symbionts by inoculation into other plant species, including grasses [48], corn [49], rice [50], and a variety of crop plants including tomato, pepper, squash, and turfgrasses [51], all of which showed improved growth and health under nutrient-limited conditions. The endophytes improved fruit yields in two varieties of tomato by approximately 2-fold. Inoculated perennial rye grass had up to a 26% increase in the foliar N content and up to a 6-fold increase in root N content [51]. Addition of the diazotrophic endophytes from wild poplar to hybrid poplars under greenhouse conditions resulted in an increased chlorophyll and total root N content. BNF in the inoculated poplar plants was estimated through ^15^N dilution to be 65% N derived from air [52]. Although these studies demonstrated the benefits of inoculation with the endophytes, it was not known if N_2_-fixation occurs in wild poplar with its natural composition and density of microbiota.

The overall objective of this study was to assess N_2_-fixation in native riparian black cottonwood (*Populus trichocarpa*) taken from its natural setting. The ^15^N incorporation assay is the most direct assay for biological N_2_ fixation since ^15^N_2_ gas is chemically inert such that tissues will only be labeled with ^15^N if the molecule was biologically reduced into usable forms. Isotopic ^15^N is present in the atmosphere at such low natural abundance compared to ^14^N (0.364 atom %^15^N) that even small increases in ^15^N label above the unexposed controls are indicative of N_2_ fixation [53]. The acetylene reduction assay (ARA), an indirect assessment of N_2_ fixation [54] was also used. We report N_2_-fixation within some wild poplar samples and demonstrate the presence of diazotrophic bacteria in these plants, helping to explain the biology of poplar as a pioneer plant species.

## Materials and Methods

### Media used

Cuttings were grown in N-free hydroponic medium (NFM) [47] or in Murashige and Skoog agar [55] modified to be N-free (NFMS; Caisson). Microbial media included the rich mannitol glutamate medium, MG/L [56], the N-limited combined carbon medium, NL-CCM, with sucrose, mannitol, and sodium lactate as C sources [57], and the N-free medium, Nfb, with malic acid as the C source [58].

### Research site and plant sampling

Plant samples were collected at the Three Forks Natural Area in King County, WA in the riparian zone of the Snoqualmie River (+47° 31' 14.30", -121° 46' 28.32"). No specific permissions were required since only small clippings were made of some of the trees in this city park; however, a permit from King County was obtained. Although some riparian zones are rich in organic N, this is not the case for this area. The substrate is river cobbles, not rich sediment, and the water is clean, originating from high alpine snow melt. The N level of the river from this site was quantified in 2011 (0.15 mg NO_3_-N and 0.30 mg NH_4_-N per liter), in 2014 (0.142 mg/L total N), and in 2015 (average of 8 samples on three sampling dates was 0.33 mg/L total N). Several branch cuttings of 7 black cottonwood (*Populus trichocarpa*) at a height of approximately 2 meters with a sample length of approximately 25 cm were collected on August 15, 2013 for the ^15^N_2_ experiments #1 and #2 and on July 17, 2015 for the ARA experiments. Samples were surface-sterilized with 10% bleach (10 min.) and 1% Iodophor (5 min.), and rinsed three times in sterile DI water. The samples collected on August 15 were cut to fit in 1-L bottles and were allowed to root in in sterile NFM for 11 days. For ^15^N experiments, samples were then cut into 5–8 cm sections, with samples from five of the seven trees healthy enough for experimentation.

### ^15^N_2_ dosing

Cylinders of compressed ^15^N_2_ isotope gas (98 atom %) were obtained from Sigma Aldrich (lot number SZ1670V). Following transfer by water displacement to serum bottles, the gas was treated with 1 ml of HCl (1N) in order to precipitate any possible trace amounts of ammonia.

### Nitrogen-free hydroponics (^15^N_2_ Experiment 1)

Rooted apical cuttings were transferred to 125-ml flasks containing 25 ml NFM and sealed with screw top septum valve caps (Mininert). Three flasks each containing two rooted cuttings were used for each individual tree. Two of these flasks were dosed with ^15^N_2_ and one was not dosed. For example, 1–1 and 1–2 shared a dosed flask and 1–3 and 1–4 shared a dosed flask, and two undosed plant samples shared a flask as well. Plants were initially exposed to 1% ^15^N_2_ gas by removing 1 ml of air and replacing it with 1 ml ^15^N_2_ gas. After 4 days, the flasks were opened inside a sterile hood for fresh air exchange for 15 minutes before being re-dosed with 5 ml of ^15^N_2_ isotope gas. A week later the flasks were aired out again and the 25 ml of NFM was replaced with fresh medium. The plants were then dosed a final time with 5 ml of ^15^N_2_ isotope gas. After 4 more days of growth, the plants were removed and prepared for mass spectrometry analysis. Overall, the plants were exposed to an experimental atmosphere with a ^15^N atom percent excess of 6.17% for 2 weeks. To verify that CO_2_ levels were adequate in the 125-ml flasks for the plant experiments, surface-sterilized cuttings of wild poplar tree 4 were incubated as described above. CO_2_ concentrations were measured for two time points a week apart at the end of the light cycles (235 and 280 ppm) and at the end of the following dark cycles (325 and 361 ppm). These values indicated that the plants were actively photosynthesizing but that the system was closed such that adequate CO_2_ levels were maintained.

### Nitrogen-free agar (^15^N_2_ Experiment 2)

For a longer study, agar rather than hydroponics, was used. Four cuttings of each of the samples from three of the poplar trees (Poplar 1, 5, and 6) were made and were transferred individually into 125 ml flasks containing 50 ml of NFMS agar. This allowed for 3 dosed and 1 undosed control plant from each tree. Plant samples were given an initial dose of 5 ml of ^15^N_2_ gas. After 8 days the flasks were opened and aired out in a sterile hood as in Experiment 1. A final dose of 5 ml ^15^N_2_ was given, and the plants were allowed to grow for 3 more weeks before being prepared for mass spectrometry as before. Throughout the experiment, the plants were exposed to an experimental atmosphere with a ^15^N atom percent excess of 6.61% for 1 month. During this one month experiment, several of the plants died including the undosed samples of Poplar1 and Poplar6 as well as one dosed sample each of Poplar1 and Poplar 5.

### *In vitro*-propagated poplar in nitrogen-free hydroponics (^15^N_2_ Experiment 3)

After obtaining shipment of Nisqually-1 tissue culture plants (provided by Steve Strauss, Oregon State University), the plants were dosed as in Experiment 1 for the wild poplar. Three rooted *in vitro*-grown plants were transferred to 125-ml flasks with 25 ml NFM and exposed to the same ^15^N_2_ dosing regime as in Experiment 1. As ^15^N incorporation controls for this experiment, *Saccharomyces cerevisiae*, *Azotobacter vinelandii* [59], and *Rahnella* sp. WP5 [47] cultures were also tested using the same serum bottle of ^15^N_2_ gas. These microbial cells had average delta ^15^N values of 47 ‰, 5075 ‰, and 142 ‰, respectively. Sterility of *in vitro*-propagated Nisqually-1 plants was tested as follows: one of the rooted cuttings was weighed and homogenized in 5 ml NL-CCM per gram of tissue, and 100 μl of diluted and undiluted extract were plated on three types of media (MG/L, NL-CCM, and Nfb) and incubated at 30°C for three days. There was no visible microbial growth. In addition, genomic DNA was prepared from two aliquots of *in vitro grown* Nisqually-1 tissue and from soil-grown Nisqually-1 and subjected to PCR using primers that amplify differentially-sized 16S rDNA fragments for mitochondria (1090 bp) and bacteria (735 bp) [60]. While PCR of the soil-grown plant sample resulted in both bands, the two tissue culture plant samples had only the mitochondrial band (data not shown), supporting the hypothesis that these plants did not harbor bacteria.

Data from two hydroponic experiments with comparable conditions (Experiment 1 and 3) were pooled for ANOVA to test the differences in δ^15^N‰ between wild-dosed, sterile-dosed, and undosed (wild and sterile combined) plants. The δ^15^N‰ values from different parts (subsamples) within a plant were averaged and the means for individual plants were used to represent each source plant as an experimental unit (replicate). The least squares means (LSMEAN) were used to make pairwise comparisons between treatments using SAS software (ver. 9.4, SAS Institute, Cary, NC).

### Isotope-Ratio Mass Spectrometry

Samples were flash frozen using liquid N_2_ and ground to a fine powder with a mortar and pestle. The samples were then transferred to aluminum weigh boats and dried at 75°C. Tin capsules (Costech) were prepared for analysis with 9–11 mg of tissue, and sent to the Alaska Isotope Lab at the University of Alaska-Fairbanks (http://ine.uaf.edu/werc/asif/) for elemental analysis isotope-ratio mass spectrometry (EA-IRMS).

### Analysis of Isotope-Ratio Mass Spectrometry Data

Data from EA-IRMS was received in the form of δ^15^N‰ (at-air). For calculation:

δN15‰=(RsamRref−1)×(1000‰)

Where R=At%N15At%N14

This can be rearranged to calculate the proportion of ^15^N to total N:

N15sam atom%=N15samTotal Nsam%=Rstd+(Rstd)×(δN15‰)1000‰1+Rstd+(Rstd)×(δN15‰)1000‰×(100%)

%Ndfa (Percent Nitrogen derived from atmosphere) is calculated [61]:

%Ndfa=N15sam atom%−N15ref atom%N15exp atmosphere atom% excess×(100%)

Where N15ref atom% is the average N15sam atom% value of the undosed control plants.

### Acetylene Reduction Assay (ARA)

For the ARA, Poplar 6 branch samples were used immediately after the July 17, 2015 collection. Branches were cut into 1 gram (0.9–1.2 g) samples of the stem portions, six from each branch, were weighed and transferred aseptically into sterile, 20 ml round bottom, beveled edge glass vials containing 1 ml filter-sterilized NFM, and sealed with magnetic caps. For nine of the samples (three from each branch), 1.8 ml of headspace gas were removed and replaced with acetylene gas (99.6% purity, Praxair). Samples were incubated for 3d in a growth chamber (Percival CU41Lx) at 25°C with a 12 hour light cycle. Headspace gas was analyzed on a gas chromatograph (TRACE GC ULTRA, Thermo Scientific, Waltham, MA) equipped with a flame ionization detector (FID) and a HayeSep R column (2.6m x 1/8” x 2.0mm). The oven temperature was set at 70°C with a flow rate of 35 ml/min. We used high purity N_2_ (g) as the carrier gas, H_2_ (g) as the fuel gas ((H_2_ generator, Parker Dominic Hunter, Cleveland, OH), and high purity synthetic air as the oxidizing gas.

A standard curve of ethylene concentrations was generated and used to calculate ethylene production. Values were adjusted for small differences in sample mass.

### Analysis of nitrogenase gene diversity within wild poplar

In parallel with the ^15^N_2_ experiments, the presence of endophytic nitrogenase gene sequences was assessed. Leaf and stem samples of surface-sterilized wild poplar trees 1–7 that had been collected in summer 2013 and grown in NFM were tested by PCR for the presence of putative diazotrophic bacteria. Polygenomic DNA was extracted using the MasterPure Plant Leaf DNA Purification Kit (Epicentre), following the manufacturer’s protocol except that 100 mg of plant tissue was used. PCR was performed using *Populus ralf* 6 primers [62] in Epicentre Fail-Safe PCR pre-mix G as a positive control for PCR. The *nifH* b1 primers [63] were used in Epicentre Pre-mix E to detect the presence of the nitrogenase subunit gene. Genomic DNA extracted from *Azotobacter vinelandii* was used as a positive control for *nifH* and no-DNA samples were included in both the *ralf* and *nifH* PCR experiments. Two additional surface-sterilized plants of wild poplar genotype 4 from the 125-ml flasks of NFM were analyzed for total diazotrophic bacteria diversity. Genomic DNA was extracted from the plants (included leaf, stem, and root together) using the MasterPure Plant Leaf DNA Purification Kit (Epicentre). PCR was performed using *nifH* b1 primers as described above. The *nifH* amplicons (351 bp) were cleaned using Exo-SAP-IT (Affimetrix) and tagged with Illumina sequencing primers with a unique ID for each sample following the standard Illumina protocol for amplicon library preparation. The libraries were then sequenced on an Illumina MiSeq sequencer using a 2x300 bp read kit. The resulting reads were processed in Geneious 7.1 (Biomatters Software LTD). The algorithm used was Geneious’ built-in “Trim and Filter” workflow with the following parameters—Error Probability limit 1%, trim the 5’ and 3’ ends, filter to retain sequences >350bp in length. Paired MiSeq reads were overlap aligned using PEAR [64] with the default settings. Based on the FASTQ values, 44,746 assembled reads were then trimmed to remove any base with an estimated error rate of greater than 1% and filtered for reads that were less than or equal to 350 bp post-trimming. This produced 6573 assembled high quality reads that spanned most of the *nifH* amplicon. A subsample of 192 of these were translated in all 6 reading frames and the correct reading frame for each read was determined by alignment against a *nifH* consensus sequence that spanned the amplicon region. The consensus sequence was created from the 2012 *nifH* database [65]. The translated sequences were then subjected to multiple sequence alignments along with *nifH* protein sequences in GenBank selected from known species and selected to span much of the *nifH* phylogenetic tree [66]. After alignment, a phylogenetic tree was constructed. Both the multiple sequence alignment and tree construction was done within Geneious using Geneious’ own alignment and tree building algorithms. Multiple sequence alignment was performed in Geneious using the MAFFT algorithm [67]. The default parameters were used (e.g. a BLOSUM62 scoring matrix, with a gap open penalty of 1.53 and an offset value of 0.123). Tree building was performed using the Geneious Tree Builder with the default parameters (e.g. a Juke-Cantor genetic distance model, and neighbor-joining with no outgrip). After tree construction, a representative sequence was selected from regions within the tree for which there was no nearby named reference sequence and searched against GenBank using BLAST [68] to identify additional reference sequences. These were then added to the alignment, and the alignment and tree was rebuilt.

### Assessment of culturable endophytic population variability

A stem cutting harvested from wild Poplar 4 in August 2014 was surface-sterilized and maintained in NFM in a 1L glass vessel. A rooted shoot that grew from the original stem cutting was harvested in 25–50 mg sections and homogenized in 5 ml NL-CCM per gm tissue for one minute. A 1:1000 dilution of each extract was prepared and 100 μl were plated onto three types of medium with varied N levels and C sources to assess microbial populations: MG/L, NL-CCM, and Nfb. Colonies were counted after 2d and 4d at 30°C. In addition, the poplar samples used in the ARA in 2015 were examined similarly for culturable endophytic populations.

## Results

### ^15^N_2_ Incorporation

**Fig 1:**
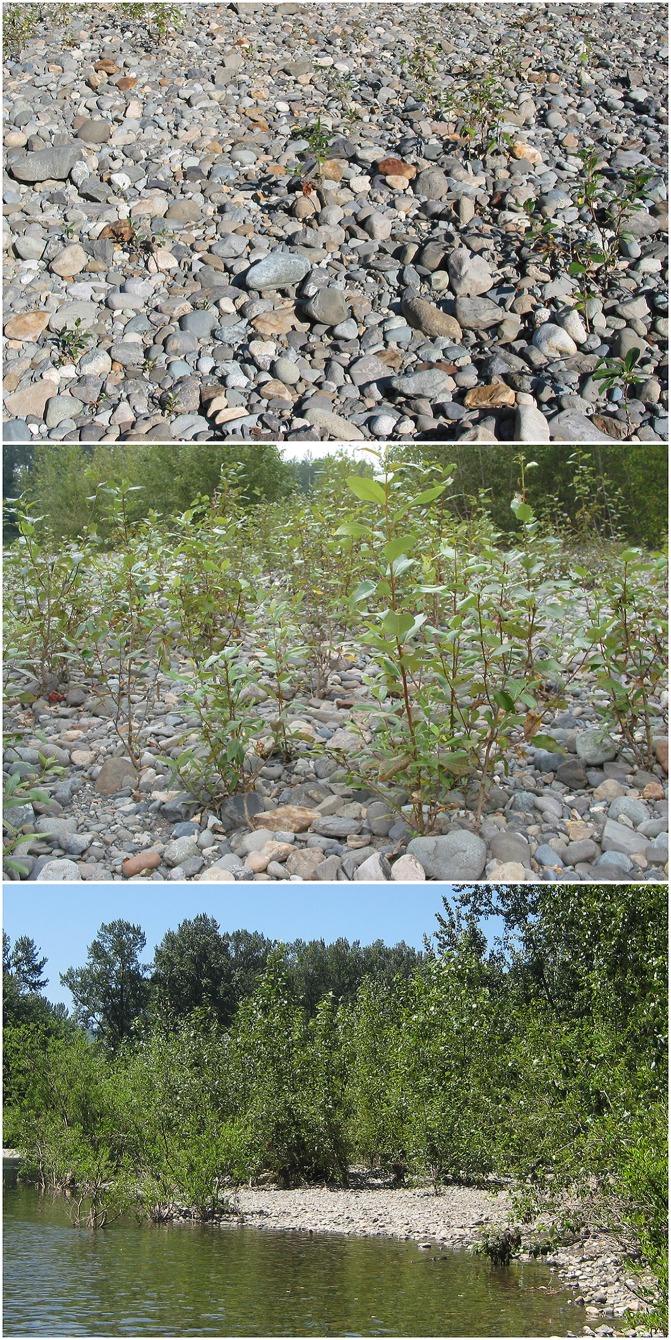
Study site along the Snoqualmie River in western Washington State. Poplar and willow are the dominant plant species in this N-limited, cobble-dominated floodplain. Photographs of the site were taken in 2002, 2006, and 2015.

**Fig 2:**
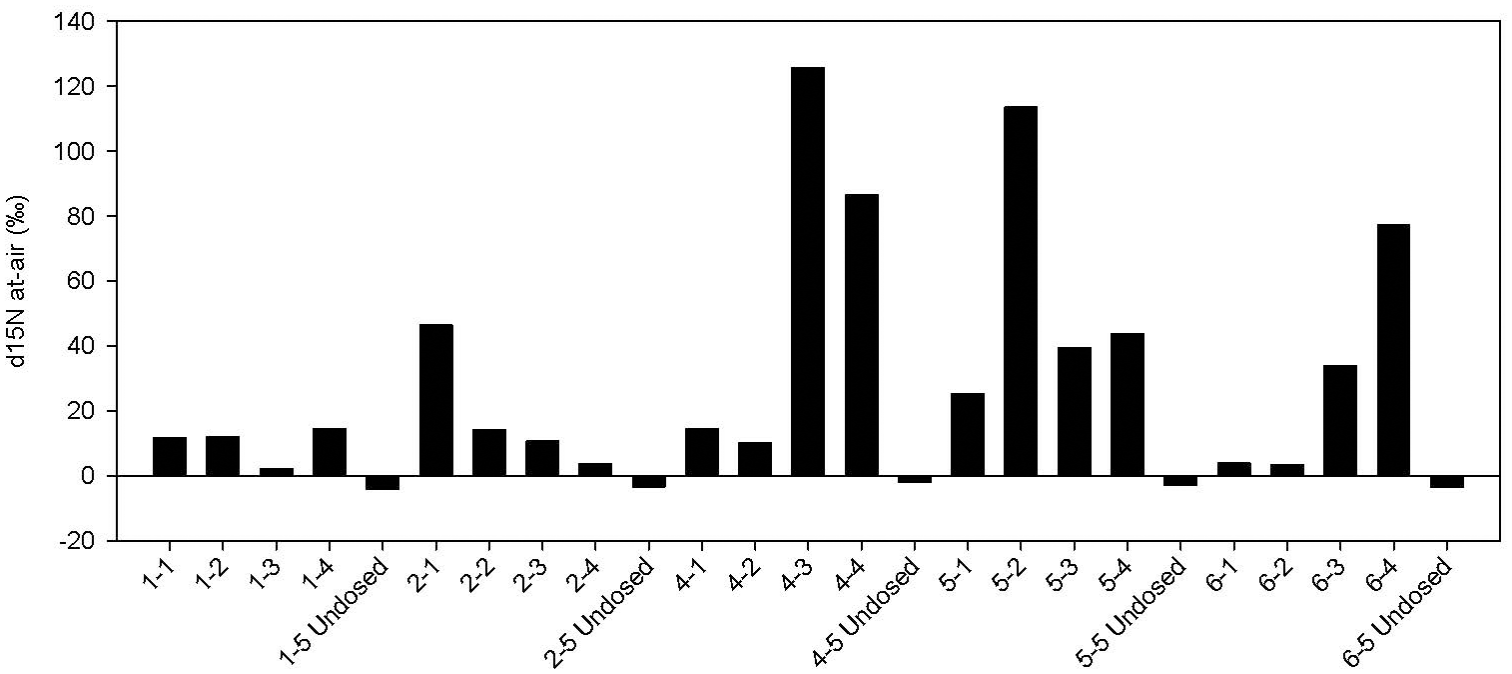
^15^N incorporation in cuttings from wild poplar plants grown in hydroponics. Cuttings from five independent plants were collected during the peak growing season in 2013. Rooted cuttings were exposed to 6.17% atom excess of ^15^N_2_ gas in N-free hydroponic medium for two weeks. Data from samples of the same plant were not averaged since the diazotrophic endophytes are not equally distributed throughout the plants.

**Fig 3:**
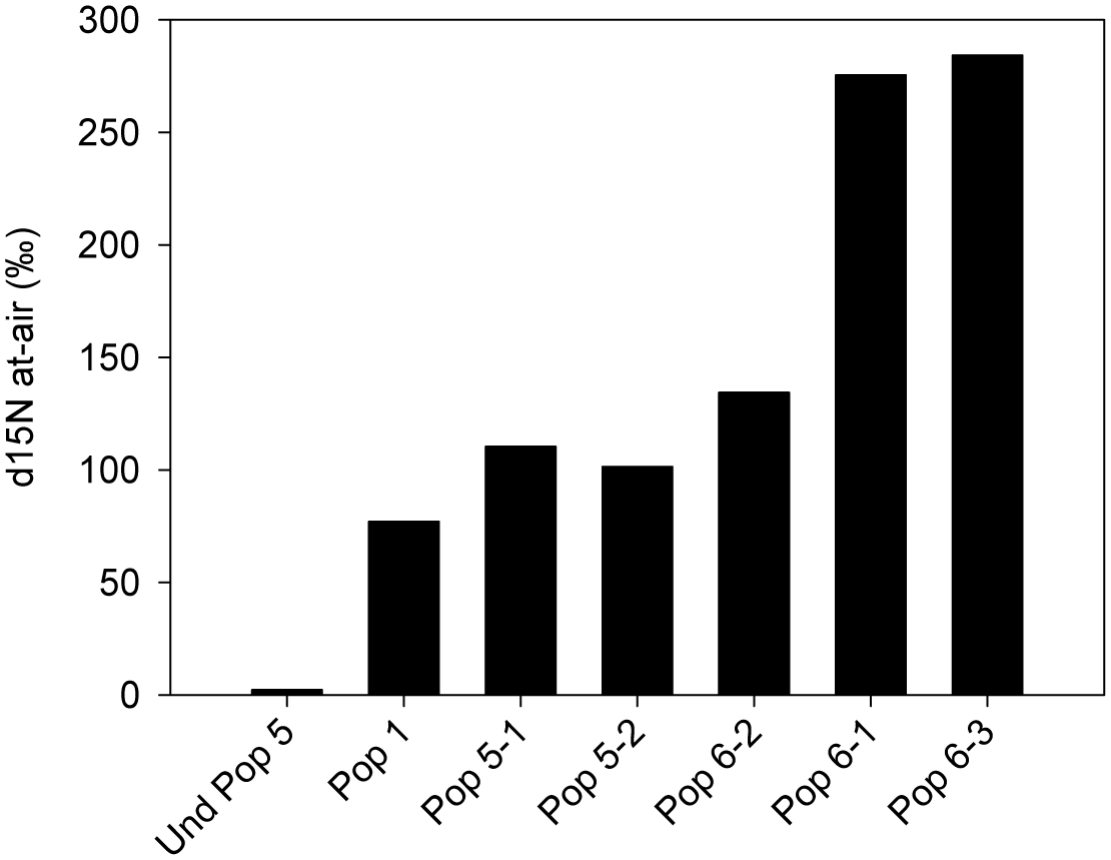
^15^N_2_ incorporation in cuttings from wild poplar plants grown in N-free agar. Cuttings from three independent wild plants (Poplar 1, 5, and 6) were collected during the peak growing season in 2013. Rooted cuttings were exposed to 6.61% atom excess of ^15^N_2_ gas for 4 weeks.

**Fig 4:**
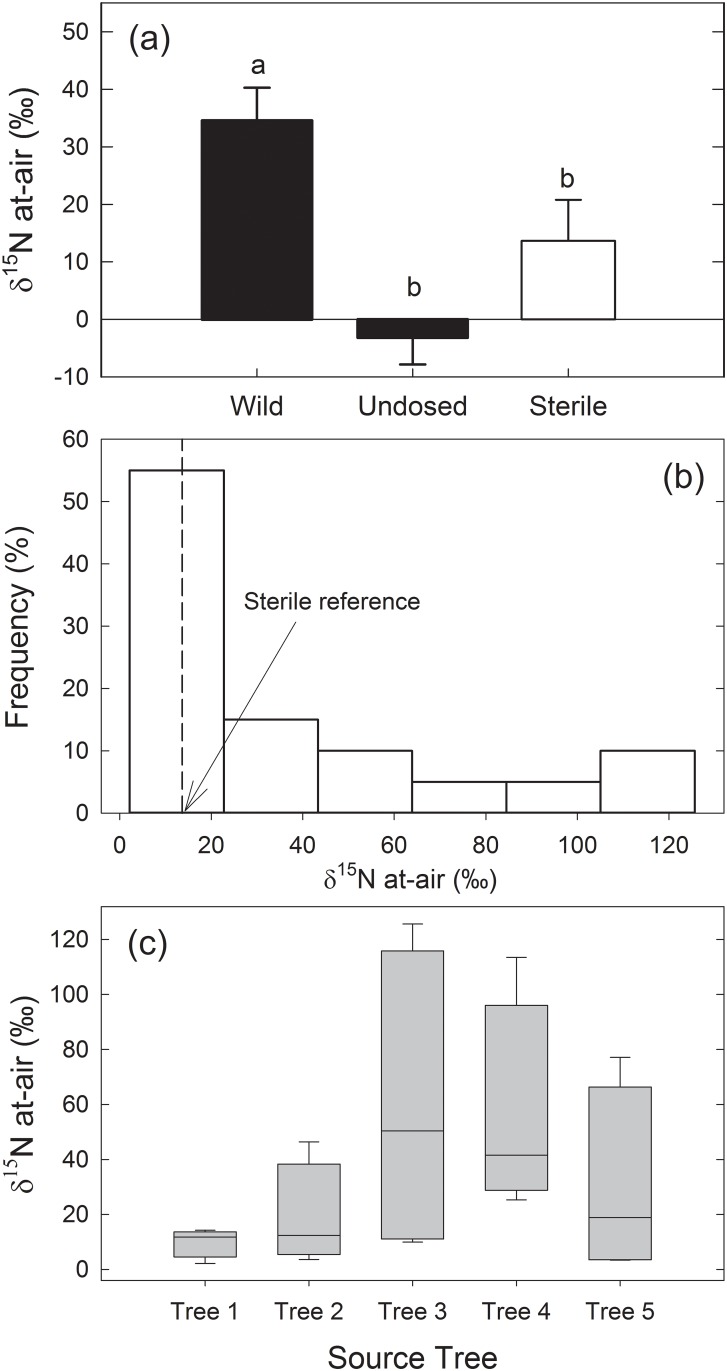
**(a)** Combined data from the hydroponic experiments (Experiment 1 and Experiment 3) illustrate an overall significantly greater ^15^N incorporation by dosed wild poplar plants compared to undosed plants (p < 0.001). *In vitro* propagated Nisqually-1 plants showed an intermediate response that was significantly lower than dosed wild plants (p < 0.05) while only marginally greater than undosed wild plants (p = 0.08). In these experiments, rooted cuttings were exposed to ^15^N_2_ gas in N-free hydroponic medium (NFM) for two weeks. Bars annotated with the same letters are not significantly different from each other based on pairwise comparisons of least squares means at alpha = 0.05. Error bars represent one standard error. **(b and c)** Distributions of δ^15^N‰ data from dosed wild poplar cuttings tested in Experiment 1. The percent frequency plot **(b)** shows that about a half of dosed wild cuttings incorporated ^15^N at similarly low levels as the *in vitro* grown Nisqually-1 cuttings from Experiment 3 (vertical dashed line). The box plot of δ^15^N‰ grouped by source trees **(c)** suggests that the capacity to harbor N_2_ fixing endophytes is likely to vary not only between trees but also between locations within a single tree.

A site along the Snoqualmie River, a natural river system in Western Washington State, was chosen for the collection of wild poplar cuttings because the water carries a very low nutrient load, and the cobble substrate is also nutrient-limited (Fig 1), yet poplar and willow plants thrive in this environment. Isotopic analysis of the poplar tissues from the two independent ^15^N_2_ labeling experiments demonstrated high levels of N_2_ fixation in some of the cuttings from wild plants, with several having δ ^15^N values over a 100 ‰ (Fig 2) and one as high as 284 ‰ (Fig 3). Overall, the ^15^N incorporation was higher in ^15^N_2_-dosed wild plants compared to undosed controls with a statistically highly significant difference (p < 0.01; pairwise comparison for H_0_: ^15^N dosed = undosed) (Fig 4a). *In vitro* propagated *P*. *trichocarpa* (clone Nisqually-1) plants were tested to verify that the ^15^N incorporation correlated with the presence of microbiota (Experiment 3). This clone was verified to be internally-sterile by microbiological and molecular tests (data not shown). The average δ ^15^N value was only 13.7 for these plants. Even when all the wild poplar hydroponic experiment data were combined, the values for ^15^N incorporation by wild poplar plants was significantly greater than that of these empirically sterile Nisqually-1 plants as well as that of un-dosed control plants (p < 0.05) (Fig 4a).

Given the variability of N_2_-fixation within the wild poplar plants, it is not possible to accurately extrapolate from the data to whole-tree estimates of N acquired through biological N_2_-fixation by the diazotrophic endophytes, but general estimates may be calculated. Levels of ^15^N in tissue suggest that during the two week period of exposure, up to 0.8% of total plant N was derived from atmospheric N_2_-fixation (Table 1, Poplar sample 4–3). As in other studies of endophytic N_2_-fixation, such rates of N accumulation per unit time are generally lower than those fixed in nodules associated with leguminous plants [7]. The poplar plants exposed to ^15^N_2_ for two weeks had a rate of N_2_-fixation of up to an average of 20.6 mg N/kg/day, if it is assumed that N_2_-fixation occurred uniformly throughout the 2-week period. We did not replace all the natural air with ^15^N_2_ and helium or argon as in other studies [18,31,69] but only spiked the headspace with the labeled gas. Given that the plant sizes were small, there was no visible growth of the plant sample during the assay, and as there may have been an enzymatic preference for the more abundant ^14^N in the system over ^15^N, the observed rate is likely an underestimate of the N_2_ fixation rate considering that under controlled greenhouse conditions, BNF in inoculated poplar was estimated to be 65% N [52].

**Table 1:** N_2_-fixation (%Ndfa) in poplar plants from five wild poplar trees.

Sample	% Ndfa	Rate of N_2_-Fixation (mg/kg/day)
1–1	0.087	2.70
1–2	0.089	3.01
1–3	0.031	0.75
1–4	0.102	2.21
1–5 UNDOSED	BDL^a^	BDL
2–1	0.292	13.2
2–2	0.101	4.55
2–3	0.081	3.04
2–4	0.040	1.48
2–5 UNDOSED	BDL	BDL
4–1	0.103	5.74
4–2	0.077	2.91
4–3	0.761	20.6
4–4	0.529	14.3
4–5 UNDOSED	BDL	BDL
5–1	0.168	5.54
5–2	0.689	14.6
5–3	0.241	11.4
5–4	0.276	11.1
5–5 UNDOSED	BDL	BDL
6–1	0.041	2.08
6–2	0.038	1.21
6–3	0.218	10.0
6–4	0.474	16.9
6–5 UNDOSED	BDL	BDL

Nitrogen derived from a ^15^N-enriched atmosphere, %Ndfa represents the proportion of total N content originating from atmospheric N-fixation, calculated using the average of the undosed samples as a reference value. Rate of N_2_ fixation is measured as mg dry weight of newly fixed N per kg of total plant tissue per day. Data were from Expt. 1.

^a^ BDL, Below detection limits

### Assessment of the diazotrophic endophytic population

Variable degrees of N_2_ fixation within cuttings from the same plant could be due to N_2_-fixing endophytes being unevenly distributed throughout the plant. Although the hypothesis that specific microorganisms were required for effective N_2_-fixation could not be tested directly at this time, we confirmed the general presence of diazotrophic endophytes in all the wild poplar trees used in the studies by conducting PCR of the genomic DNA to amplify the nitrogenase subunit gene, *nifH*. All of the wild poplar plant samples were positive for the presence of the *nifH* gene (S1 Fig) indicating that endophytic or closely associated diazotrophic microorganisms were present. To assess the culturable endophytic population within the wild poplar cuttings, we homogenized three surface-sterilized samples that were of similar size to those used in the ^15^N_2_ assays and then plated the extracts on N-limited medium. The total population density ranged from 5.1 x 10^6^ to 1.9 x 10^7^ CFU/g. Sequencing of the *nifH* amplicons from one of the wild poplar trees revealed a diversity of diazotrophic taxa (S2 Fig). These included *nifH* sequences from genera we had previously isolated and studied including *Burkholderia* and *Sphingomonas* but also others such as *Azospirillum brasilense* and *Bradyrhizobium sp*., photosynthetic diazotrophs such as *Rhodospirillum rubrum*, *Rhodobacter capsulatus* and *Nostoc punctiforme*, and Archaeal species including *Methanococcus maripaludis* and *Methanosarcina acetivoran*. Many of the other sequences most closely matched *nifH* sequences of uncultured bacteria in the database. A detailed assessment of the microbiome of these poplar trees is underway (U.S. Department of Energy Joint Genome Institute).

**Fig 5:**
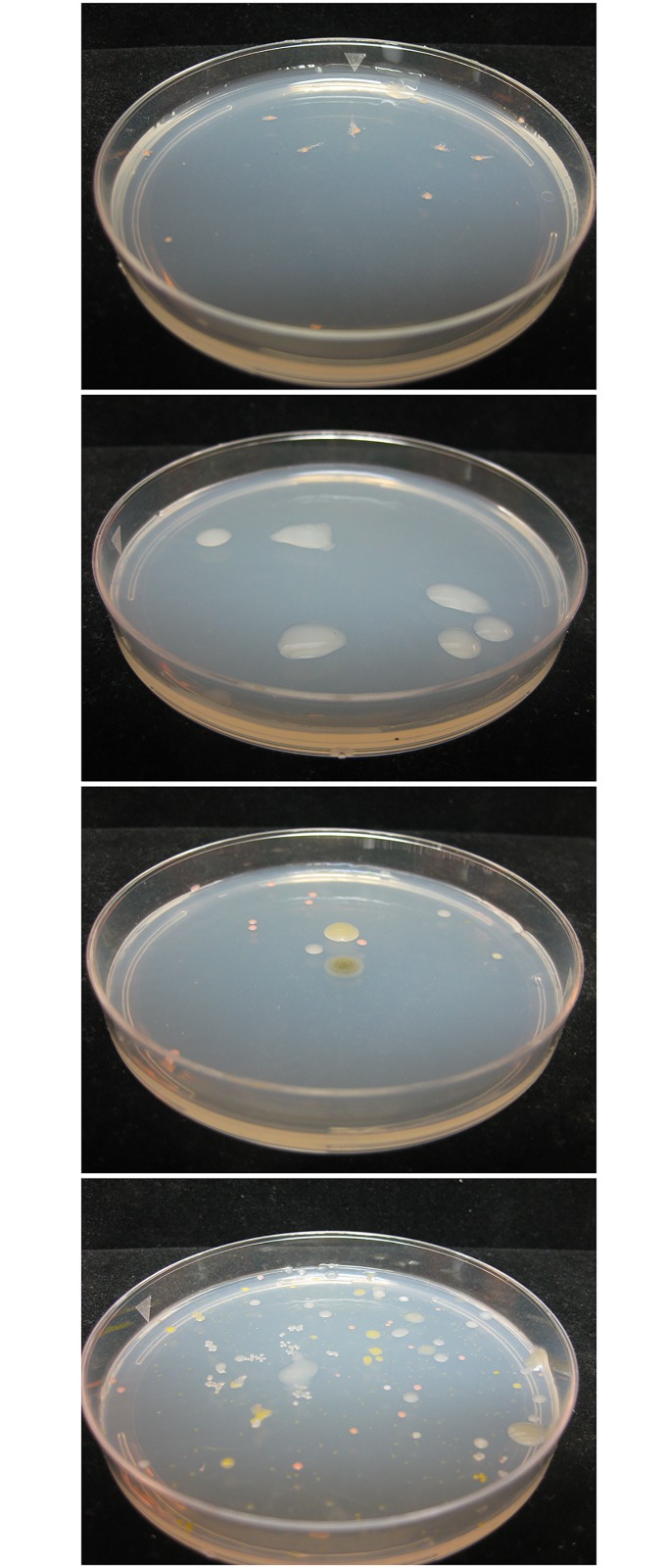
Variability in endophytic population and composition in four stem sections of wild poplar genotype 4, each extracted with 5 ml/gm. Extracts were diluted 1:1000 and 100 μl plated on N-limited combined carbon medium (NL-CCM). Plates were photographed after 4 days at 30°C.

Having confirmed that the wild poplar trees harbored diazotrophic microbial species, we assessed microbial diversity within different sections of the same tree. Wild Poplar 4 was chosen for this study since, in the previous growing season, some of the cuttings of this tree had exhibited high ^15^N incorporation (samples 4–3 and 4–4) while others had exhibited no significant incorporation (samples 4–1 and 4–2). Both the cultured endophytic population density (Table 2) and composition (Fig 5) varied greatly in different sections of the same plant. For example, the colony forming units (CFU) on N-limited medium differed by 2 orders of magnitude in closely adjacent stem samples (samples 3 and 4). The colony morphology differed considerably (Fig 5) suggesting that species diversity was different even in adjacent samples of the same tissue.

**Table 2:** Variability of the culturable endophytic population of Wild Poplar 4.

Sample	Tissue	CFU on MG/L	CFU on NL-CCM	CFU on Nfb
1	Root	2 x 10^7^	1.7 x 10^7^	8.2 x 10^6^
2	Root	3.4 x 10^7^	TNTC	2.0 x 10^7^
3	Stem	5.7 x 10^6^	1.3 x 10^7^	2.9 x 10^6^
4	Stem	4.0 x 10^5^	7.5 x 10^5^	4.5 x 10^5^
5	Stem	3.0 x 10^5^	7.0 x 10^5^	3.5 x 10^5^
6	Stem	2.9 x 10^6^	5.5 x 10^5^	1.1 x 10^6^
7	Leaf	6.4 x 10^6^	4.3 x 10^6^	1.1 x 10^6^
8	Leaf	1.5 x 10^7^	1.7 x 10^7^	5.7 x 10^6^
9	Leaf	1.0 x 10^6^	7.0 x 10^5^	2.0 x 10^5^
10	Leaf	TNTC	1.4 x 10^6^	4.5 x 10^5^

Culturable endophytes were extracted in 5 ml NL-CCM per gm of tissue. Extracts were diluted 1:1000 and 100 μl were plated on three types of medium. The number of colony forming units (CFU) was assessed after 4 days. TNTC, too numerous to count.

### Acetylene reduction assay

As a further test of N_2_ fixation in wild poplar, we used the acetylene reduction assay [54]. This commonly used indirect measure of N_2_ fixation has been used to study endophytic and associative N_2_-fixation, and relies on the ability of the nitrogenase enzyme to reduce acetylene gas to ethylene which can be measured by gas chromatography [18,25,70–72]. We chose Wild Poplar 6 for this study as it had the highest level of N_2_-fixation (Fig 3) although this level was variable (Fig 2). Branch samples from Wild Poplar 6 were collected in the following growing season, surface-sterilized, and transferred to GC vials. Half of the samples were dosed with acetylene, and incubated for three days. The undosed stem samples produced an average of only 5 mmol ethylene per gm (Table 3), indicating the background levels of ethylene production by the plants. Of the nine dosed samples, three had levels of ethylene production that were 6, 12, and 21-fold higher than the averaged undosed samples. The other six samples had values comparable to the undosed samples.

**Table 3:** Acetylene reduction assay results of Wild Poplar 6 stem samples.

Plant Sample	mmol ethylene per gram
Undosed 1	4.84
Undosed 2	3.38
Undosed 3	4.50
Undosed 7	4.52
Undosed 8	5.01
Undosed 9	3.52
Undosed 13	4.42
Undosed 14	7.34
Undosed 15	8.46
Dosed 21	20.63
Dosed 22	61.42
Dosed 23	6.19
Dosed 27	8.52
Dosed 28	5.76
Dosed 29	9.05
Dosed 33	5.69
Dosed 34	4.24
Dosed 35	106.9

Samples were incubated for 3d before headspace analysis by gas chromatography. Ethylene was calculated using a standard curve and adjusted for plant mass.

**Fig 6:**
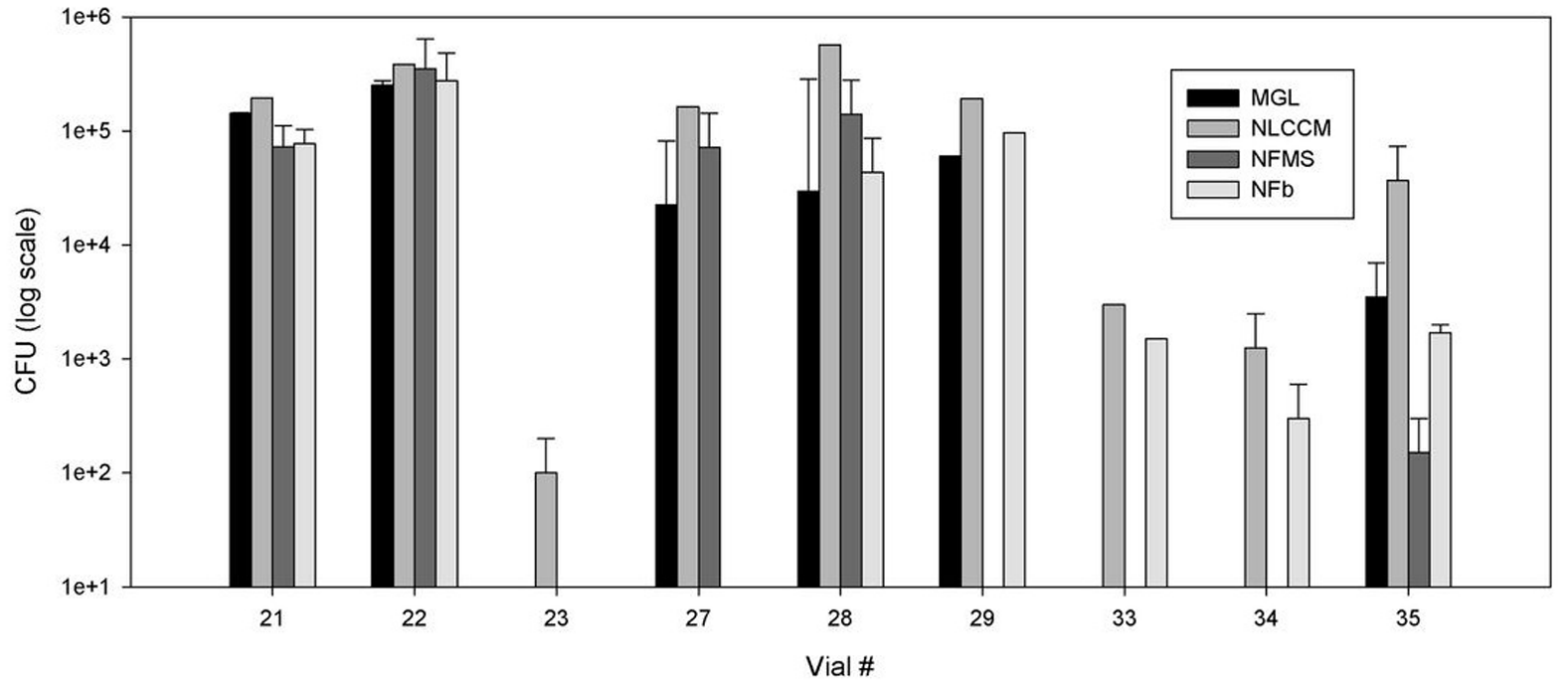
Variability of the culturable endophytic population of wild poplar genotype 6 stem samples used in the acetylene reduction assay experiment. CFU, colony forming units.

The high variability in N_2_-fixation in wild poplar, even within the same tree, was therefore determined using two independent assays in two different growing seasons. The samples used in the ARA were tested for culturable endophyte populations at the end of the experiment. The population densities were highly variable in the different stem sections (Fig 6). There was no correlation between overall number of culturable bacteria and ARA values.

## Discussion

The data presented here represent the first direct demonstration that N_2_-fixation is possible within wild poplar trees and the first demonstration that a varied diazotrophic microbial population exists within individual poplar sections. Further studies are needed to localize *in planta* active diazotrophic endophytes and the dynamics of N transfer between these potentially symbiotic partners. The variation in N_2_-fixation amongst different cuttings of the same tree alludes to the intriguing possibility that there may be microbial social requirements that limit effective N_2_ fixation to particular groupings within the plant that have achieved a threshold of density [73] or diazotrophic species composition. Since the ARA results did not correlate with the overall numbers of cultured bacteria, it is likely that there are either specific diazotrophic strains that are required for effective *in planta* N_2_-fixation or that these strains were not culturable on the media tested. This population of diazotrophs would be of great importance but would not be seen in our CFU counts. Using *nifH* gene expression analysis in sugarcane, it was shown that rhizobial species rather than the dominant cultured endophytic species were likely the primary contributors of N [74,75]. Likewise, a metagenomic study of the endophytic community in rice revealed that there was a high apparent density of N-fixing endophytes but that the dominant *nifH* expressed was only that of *Rhizobium* [8]. Molecular studies are required to identify the active diazotrophic species and their density requirements for effective N_2_-fixation *in vivo* in *Populus*. Such studies including fluorescent *in situ nifH* hybridization and nanoscale secondary ion mass spectrometry (NanoSIMS) were recently approved by the U.S. Department of Energy Joint Genome Institute and will begin in 2016.

While there have been several studies quantifying N_2_-fixation in grasses, the variability in N_2_-fixation such as we have encountered in *Populus* has not been reported. The structure of poplar trees, and eudicots in general, is much more complex than that of monocots. The trunk and branches, internodes and nodes, leaves and petioles of eudicots—all provide unique habitats that may lead to a highly variable microbiota occupying the different niches. Each of these compartments is likely influenced within trees by its location in the canopy, the sun exposure, and the age of the leaf or branch. There are multiple factors involved in microbial community assembly including plant genotype, environment, microbe-microbe interactions [76] and the season [77]. Plants seem to have some influence over the microbial population of the rhizosphere [78] as well as the endosphere [44]. There may also be a general selection for particular functional traits [79]. In the N-limited site used in this study, diazotrophy is likely a selected trait. The culturable endophytic population of the poplar and willow trees is dominated by diazotrophic bacteria of many different species [47]. A culture-independent assessment of the microbiome of the leaves of one poplar tree revealed a high percentage of *Burkholderia* (24%) and *Sphingomonas* (16%) (unpublished data), genera that include many N-fixing species. In the *nifH* analysis presented here, a broad range of diazotrophic species was represented including the well-studied plant-associated diazotrophs, *Azospirillum brasilense* and *Bradyrhizobium* sp. Where the different strains ultimately reside within the available niches of an entire tree, and in what microbial communities and densities, are likely not homogeneous throughout the plant. Therefore, unlike the legume-rhizobium symbiosis in which the N_2_-fixing bacteria are housed in specific nodule structures, diazotrophic endophytic bacteria are unevenly distributed, and could lead to variation in N_2_-fixation in different areas of trees.

The ^15^N incorporation assay is viewed as the most direct method for assessing N_2_-fixation compared to the ^15^N dilution assay or isotopic differences in natural abundance. A criticism of the technique, however, is that there is possible contamination of ^15^N-labeled ammonia or nitrate in the ^15^N_2_ gas [80]. Treatment of the gas with HCl precipitates out any contaminating ammonia, and since only the gas is delivered to the samples, any contaminating nitrate would not be present. Since the sterile plants had the lowest ^15^N incorporation and since plants in the same flask (Expt 1) had different ^15^N incorporation values, the HCl treatment was effective in removing any possible contamination. The ARA is commonly used to qualitatively assess endophytic and epiphytic N_2_-fixation but can have limitations [81]. One concern is that ethylene, the indicator of N_2_-fixation in this assay, is produced by the plant. However, by testing equal numbers of undosed and acetylene-dosed samples, a baseline can be established. Another method for testing for BNF is through the ^15^N natural abundance assay. This assay relies upon a comparison to substrate and to “non-fixing” controls. In our low-nutrient, cobble-dominated study site, however, the substrate is rocky and the only plants present are the poplar and willow, making this assay untenable.

The possibility of biological N_2_-fixation by endophytes has faced criticism because the nitrogenase enzyme is inhibited by oxygen and is therefore thought to require the specialized structure of root nodules. However, since free-living diazotrophic bacteria evolved long before legumes or actinorhizal plants [6], this criticism cannot be justified. N_2_-fixing bacteria can utilize a range of mechanisms to protect the nitrogenase enzyme complex [5,6]. *Trichodesmium*, for example, is an ancient marine diazotrophic cyanobacterium that fixes N_2_ effectively without the specialized heterocyst cells common to some other cyanobacteria species [82]. In this bacterium, oxygenic photosynthesis and N_2_-fixation operate concurrently during the day through temporal and spatial segregation and a reduction of photosynthetically-evolved oxygen [83] to solve the so-called “oxygen paradox” [5,84]. While the nitrogenase enzyme is inhibited by oxygen, the exceptionally high energy demands of N_2_-fixation often require oxidative phosphorylation for maximum ATP generation. By rapidly utilizing sugars, intracellular oxygen can be depleted or at least sufficiently reduced. The free-living, rhizospheric diazotroph, *Azotobacter vinelandii*, for example, can employ this respiratory protection mechanism as well as conformational protection, avoidance and spatial separation [59]. Endophytes may use a variety of strategies to achieve *in planta* N_2_-fixation. One method could be through migrating to a microhabitat that has optimal oxygen levels [85]. *nifH* gene expression is regulated by oxygen, and has been shown to be expressed *in planta* by endophytes and associated bacteria, thus demonstrating that permissive microhabitats are available or that metabolic conditions have otherwise been met [11–14]. In addition, since some of the *nifH* sequences of wild poplar matched those of anaerobic Archaeal species, there are presumably niches within the plant tissue that are anaerobic. Exopolysaccharide production is a common trait in endophytes [86–89], and could possibly provide the necessary microaerobic environment for N_2_-fixation inside biofilms. More research is necessary to elucidate the specific mechanisms used by diazotrophic endophytes to protect the nitrogenase complex.

*Populus* species have long been known as early-successional trees able to colonize highly disturbed sites. We have previously shown that poplar and willow at this study site host a variety of microorganisms within branch tissues, some of which are capable of fixing N_2_ *in vitro*. Inoculation of cultivated poplar plants with these wild poplar endophytes increased N_2_ fixation [52]. Addition of the wild poplar endophytes to grasses and crop species (eudicots as well as monocots) increased plant growth in N-limited conditions [48–51]. The host range also encompassed gymnosperms, providing increased biomass under N-limited conditions to the commercially important forest tree, Douglas-fir (*Pseudotsuga menziesii*) [90]. These cross-host studies strongly support the hypothesis that the diazotrophic endophytes of wild poplar can provide significant amounts of N to the plant host. Other research has also given indirect evidence of N_2_ fixation in poplar. Metabolic profiling of hybrid poplar inoculated with a *Paenibacillus* strain from within micropropagated poplar suggested N_2_ fixation [91]. In comparison to uninoculated controls, the inoculated poplar had increased levels of asparagine and urea, which suggests increased N assimilation. Other research in the early 1980’s indicated that N_2_ fixation may be occurring within poplar. For example, wetwood samples of eastern cottonwood (*Populus deltoides*) tested positive with the acetylene reduction assay (ARA), and the degree of acetylene reduction increased when glucose was added and decreased when NH_4_Cl was added, demonstrating that this activity was regulated as expected [92]. Wood samples of black cottonwood (*P*. *trichocarpa*) from four felled trees also tested positive with the ARA [93]. Our results provide the first direct evidence of N_2_-fixation within wild poplar. The variability in both microbial composition and concentration points to the need for a better understanding of endophytic colonization and its relation to effective N_2_-fixation. As poplar was the first tree genome to be sequenced [94] and given that genomic sequencing of the first diazotrophic endophytes isolated from poplar is now underway, the poplar-endophyte symbiosis could serve as a model system for studying N_2_-fixation in trees at the molecular level.

One of the next great challenges will be maximizing food and biomass production in a sustainable way, and innovative new technologies must be developed. While one approach has been to attempt engineering of nodulation into crop plants, a simpler approach is evident [95,96]. Tailoring of the microbiome of plants can increase plant growth with reduced inputs. Although inoculation of crop plants with specific diazotrophic strains can contribute significant levels of N, it is often still necessary to apply some N fertilizer [97]. Further research is required to identify the best inoculants, especially for specific plant genotypes [98]. Considering that long-lived trees in natural, nutrient-limited environments have had the longest time and the greatest need to select beneficial microbial strains from their environment, such pioneer tree species may therefore be the best source for effective inoculants. The microbiota of wild poplar trees helps an exceptionally broad range of plant species, from grasses [48,51] to conifers [90], to overcome nutrient deficiencies. A deeper understanding of the microbiome of this non-nodulated early successional pioneer plant species may not only provide insight on how to optimize this species' biomass production, but also provide critical insights into how microbial endophytes can increase production of other bioenergy plant species and agricultural crops while at the same time reducing reliance on chemical fertilizers.

## Supporting Information

S1 Fig PCR and sequencing of nitrogenase gene fragments in poplar tissue. Leaf and stem samples of surface-sterilized wild poplar genotypes 1–7 that had been collected in summer 2013 and grown in NFM were tested by PCR for the presence of putative diazotrophic bacteria. PCR was performed using Populus ralf 6 primers as a positive control (upper panel). The nifH b1 primers [63] were used to detect the presence of nitrogenase (lower panel). Genomic DNA extracted from *Azotobacter vinelandii* was used as a positive control for *nifH*. The arrow indicates the *nifH* gene product. S, stem; L, leaf. Lanes 1, 1Kb Plus DNA ladder; Lanes 2, no-DNA controls; Lane 3, Pop1S; Lanes 4, Pop1L; Lanes 5, Pop2S; Lanes 6, Pop2L; Lanes 7, Pop3S; Lanes 8, Pop3S.2; Lanes 9, Pop3L; Lanes 10, Pop4S; Lanes 11, Pop4L; Lanes 12, Pop5S; Lanes 13, Pop5L; Lanes 14, Pop6S; Lanes 15, Pop6L; Lanes 16, Pop7S; Lanes 17, Pop7L; Lanes 18, empty; Lanes 19, *Azotobacter vinelandii*.
(TIF)

S2 Fig Sequence analysis of the nitrogenase gene (*nifH*) population in poplar. Polygenomic (plant and endophytic) DNA was isolated from two rooted cuttings of Wild Poplar 4. A subset of the branches were labeled of those with similarity 90% or above in GenBank. Unlabeled branches indicate sequences with closest matches to *nifH* genes of uncultured bacteria in Gene Bank. The three colored groups represent Group 1 (black), Group II (red) and Group III (blue) *nifH* sequences as per [66].
(TIF)
