# Supplementary figures and images for: Variable Nitrogen Fixation in Wild Populus
Source: PLoS One. 2016 May 19;11(5):e0155979. doi: 10.1371/journal.pone.0155979 (PMC4873266; doi:10.1371/journal.pone.0155979)

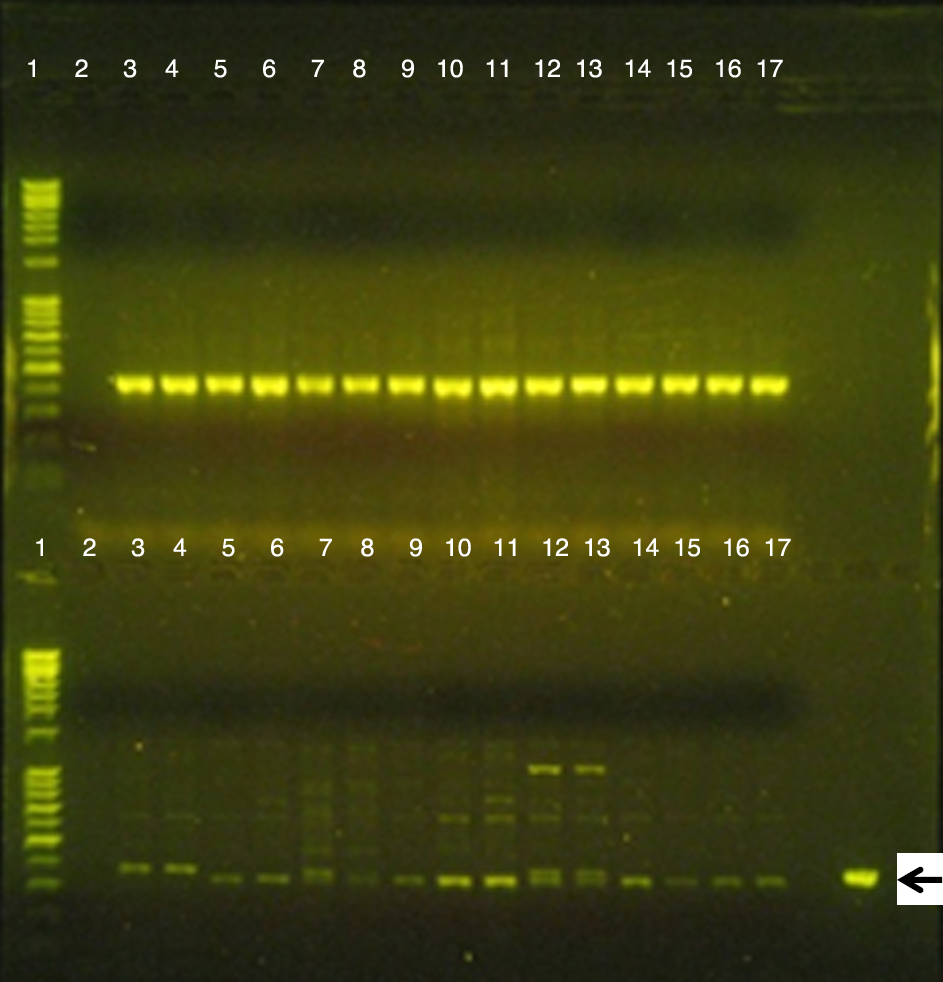

Supplement: S1 Fig — Leaf and stem samples of surface-sterilized wild poplar genotypes 1–7 that had been collected in summer 2013 and grown in NFM were tested by PCR for the presence of putative diazotrophic bacteria. PCR was performed using Populus ralf 6 primers as a positive control (upper panel). The nifH b1 primers [63] were used to detect the presence of nitrogenase (lower panel). Genomic DNA extracted from Azotobacter vinelandii was used as a positive control for nifH. The arrow indicates the nifH gene product. S, stem; L, leaf. Lanes 1, 1Kb Plus DNA ladder; Lanes 2, no-DNA controls; Lane 3, Pop1S; Lanes 4, Pop1L; Lanes 5, Pop2S; Lanes 6, Pop2L; Lanes 7, Pop3S; Lanes 8, Pop3S.2; Lanes 9, Pop3L; Lanes 10, Pop4S; Lanes 11, Pop4L; Lanes 12, Pop5S; Lanes 13, Pop5L; Lanes 14, Pop6S; Lanes 15, Pop6L; Lanes 16, Pop7S; Lanes 17, Pop7L; Lanes 18, empty; Lanes 19, Azotobacter vinelandii. (TIF) [file pone.0155979.s001.tif]

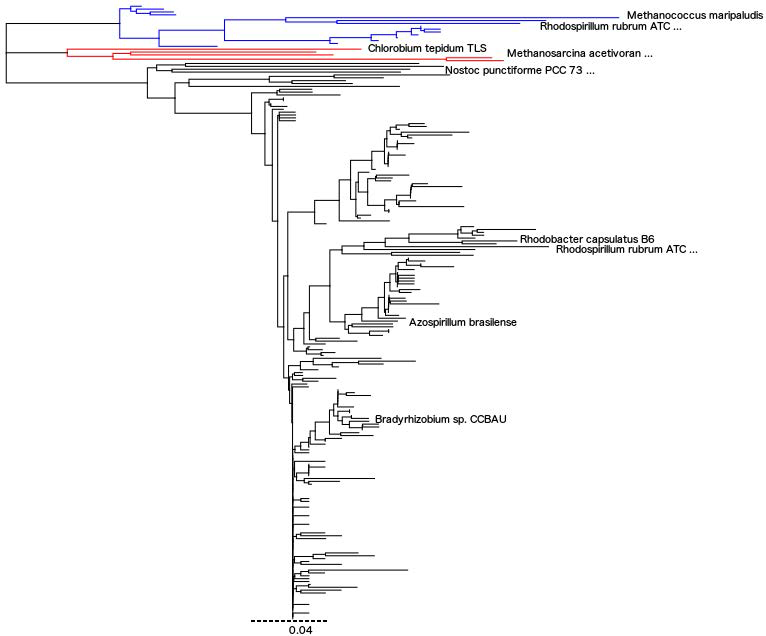

Supplement: S2 Fig — Polygenomic (plant and endophytic) DNA was isolated from two rooted cuttings of Wild Poplar 4. A subset of the branches were labeled of those with similarity 90% or above in GenBank. Unlabeled branches indicate sequences with closest matches to nifH genes of uncultured bacteria in Gene Bank. The three colored groups represent Group 1 (black), Group II (red) and Group III (blue) nifH sequences as per [66]. (TIF) [file pone.0155979.s002.tif]
